# COPD: comparative study of vaccinated and unvaccinated patients for pneumococcal disease

**DOI:** 10.36416/1806-3756/e20240369

**Published:** 2025-11-14

**Authors:** Adriana de Siqueira Carvalho Knabben, Rosemeri Maurici

**Affiliations:** 1. Somed - Instituto do Sono e Medicina Respiratória, Florianópolis (SC) Brasil.; 2. Departamento de Clínica Médica, Universidade Federal de Santa Catarina, Florianópolis (SC) Brasil.; 3. Hospital Universitário, Universidade Federal de Santa Catarina, Florianópolis (SC) Brasil.; 4. Hospital Nereu Ramos, SES-SC, Florianópolis (SC) Brasil.; 5. Programa de Pós-Graduação em Ciências Médicas, Universidade Federal de Santa Catarina, Florianópolis (SC) Brasil.; 6. Núcleo de Pesquisa em Asma e Inflamação das Vias Aéreas, Universidade Federal de Santa Catarina, Florianópolis (SC) Brasil

## TO THE EDITOR:

COPD is a heterogeneous disease and one of the top three causes of death worldwide.^1^ COPD patients may experience exacerbations, which are common and associated with decline in lung function, increased use of health resources, hospitalizations, and mortality.^2^ Pharmacological interventions help reduce exacerbation frequency, but smoking cessation and vaccination are also crucial.^1^ ^,^ ^3^ ^,^ ^4^

Pneumococcal vaccines were developed to stimulate protective antibodies against *Streptococcus pneumoniae* strains associated with pneumococcal disease.^5^ Conjugate vaccines increase immunogenicity given that polysaccharide antigens are chemically attached to a highly immunogenic inactive protein, inducing a dependent B and T response and an immune memory response.^5^ ^,^ ^6^

There are few studies comparing pneumococcal vaccines in the population of patients with COPD. For that reason, this study aimed to evaluate whether immunization with pneumococcal vaccine(s) in patients with COPD would interfere in the frequency and severity of acute events related to this disease.

In this historical cohort, patients from two centers in the city of Florianópolis, Brazil, were recruited. To be eligible for the study, patients must have been 40 years of age or older; have had a confirmed diagnosis of cigarette-smoking-related COPD: have presented with a postbronchodilator FEV_1_/FVC ratio < 0.70; have had at least a 10 pack-year history of smoking; have been vaccinated for influenza for two consecutive years, and have been on regular treatment for COPD.

This study was approved by the Human Research Ethics Committee of the institution (CAAE 13620419.6.0000.0110). We included patients who gave written informed consent and underwent the following scheme of pneumococcal vaccination:

Group PPV-23: patients vaccinated only with 23-valent pneumococcal polysaccharide vaccine (PPV-23), having been applied in a period equal to or less than 5 years prior to the inclusion in the study.

Group PPV-23+PCV-13: patients vaccinated with both pneumococcal vaccines: 13-valent pneumococcal conjugate vaccine (PCV-13) and PPV-23, regardless of sequence, and the last vaccine having been applied in a period equal to or less than 5 years prior to the inclusion in the study.

Control group: patients who have never received pneumococcal vaccine.

We excluded patients with asthma and/or chronic suppurative lung diseases, those with clinical conditions that could reduce vaccine response, such as immunosuppression, as well as those who were not under regular medical follow-up.

All participants received annual influenza vaccines during the period of the study. The study period was two years after pneumococcal vaccination(s) or in the two years prior to inclusion in the study (influenza control group).

Medical records of the patients were analyzed, considering the medical visits performed between February of 2014 and March of 2021. At the time of the consultation, the COPD Assessment Test questionnaire and the modified dyspnea scale of the Medical Research Council were performed. Confirmation of vaccination was mandatory.

The main outcome was experiencing any exacerbation of COPD. Secondary outcomes were experiencing any hospitalization (ward or ICU), health care utilization (hospitalization or emergency care, for any cause), classification of COPD according to GOLD, and adverse events to the vaccines of the study (pneumococcal and influenza).

Results were summarized as absolute and relative frequencies, means, and standard deviations. The associations were evaluated using the chi-square test at a significance level of 5%. During the study period, 205 patients were considered eligible, and 122 were included (41 in the PPV-23 group, 41 in the PPV-23+PCV-13 group, and 40 in the control group).

The mean age was 70.5 years, with a predominance of men (61.5%). The mean age of the first or only pneumococcal vaccination was 67.7 years, ranging from 46 to 86 years. The mean smoking history was 53.4 pack-years, and the majority of those patients (82.8%) were former smokers.

There was no significant difference between the groups regarding the frequency of exacerbations during the study period. In the PPV-23 group, 63.4% of the patients had at least one exacerbation. In the PPV-23+PCV-13 and control groups this frequency was, respectively, 80.5% and 70.0% (p = 0.226). In relation to hospitalizations in a ward, the control group experienced a higher frequency of hospitalizations (at least one hospitalization): 25%; PPV-23 group: 7.3%; and PPV-23+PCV-13 group: 4.9% (p = 0.011). In relation to any hospitalization (either in a ward or ICU), this difference was also significant: 25.0% in the control group, 7.3% in the PPV-23 group, and 9.8% in the PPV-23+PCV-13 group (p = 0.046). The use of health services, either due to exacerbation of COPD or decompensation of other comorbidities, did not differ significantly among the groups (Table 1).

**Table 1:** Characteristics of the COPD patients included in the study and outcomes (N = 122).^a^

Variable	Group			Total	p
	PPV-23 (n = 41)	PPV-23 + PCV-13 (n = 41)	Control (n = 40)		
Male sex	24 (58.5)	23 (56.0)	28 (70.0)	75 (61.5)	0.39
Age, years	68.90 ± 9.1	74.51 ± 8.6	68.13 ± 9.6		0.34
Age at first pneumococcal vaccine, years	65.5 ± 8.8	67.6 ± 9.3	------		0.32
Comorbidities Yes No	37 (90.0) 4 (10.0)	39 (95.1) 2 (4.9	31 (77.5) 9 (22.5)	15 (12.3) 107 (87.7)	0.04
GOLD Grade 1 Early 2 Moderate 3 Severe 4 Very Severe	1 (2.5) 16 (39.0) 20 (48.5) 4 (10)	1 (2.5) 20 (48.5) 13 (32) 7 (17)	7 (17.5) 22 (55) 9 (22.5) 2 (5)	9 (7.4) 58 (47.5) 42 (34.5) 13 (10.6)	0.01
GOLD Class A B E	12 (29.5) 14 (34.0) 15 (36.5)	9 (22) 9 (22.0) 23 (56.0)	11 (27.5) 7 (17.5) 22 (55.0)	32 (26.2) 30 (24.6) 60 (49.2)	0.37
Ward hospitalization					0.01
No	38 (92.7)	39 (95.1)	30 (75.0)	13 (10.6)	
Yes	3 (7.3)	2 (4.9)	10 (25.0)	15 (12.3)	
ICU hospitalization					0.35
No	41 (100)	39 (95.1)	38 (95.0)	118 (96.7)	
Yes	0 (0.0)	2 (4.9)	2 (5.0)	4 (3.3)	
Any hospitalization					0.046
No	38 (92.7)	37 (90.2)	30 (75.0)	105 (86)	
Yes	3 (7.3)	4 (9.8)	10 (25.0)	17 (14)	
Health care utilization					0.26
No	13 (31.7)	7 (17)	12 (30)	32 (26.2)	
Yes	28 (68.3)	34 (83)	28 (70)	90 (73.8)	
Exacerbation					0.23
No	15 (36.6)	8 (19.5)	12 (30.0)	35 (28.7)	
Yes	26 (63.4)	33 (80.5)	28 (70.0)	87 (71.3)	
Adverse effects					0.17
No	32 (78.0)	38 (92.7)	34 (85.0)	104 (85.2)	
Yes	9 (22.0)	3 (7.3)	6 (15.0)	18 (14.8)	

PPV-23: 23-valent pneumococcal polysaccharide vaccine; and PCV-13: 13-valent pneumococcal conjugate vaccine. Values expressed as n (%) and mean ± SD.

One previous study evaluated PCV-13 vaccination and its impact on COPD exacerbations.^7^ There was no significant difference in relation to the rate of exacerbations, but the absence of PCV-13 vaccination almost tripled the risk of hospitalizations.

Ignatova et al. demonstrated that the administration of PPV-23 or PCV-13 reduced by four times the chance of a COPD exacerbation in the first year of that study, when compared with the non-immunized group, and reduced hospitalizations,^8^ but PPV-23 had a progressive reduced efficacy after the first year of immunization.

Cochrane’s last systematic review of 2017 about pneumococcal vaccines in COPD patients have shown that vaccination significantly reduced the probability of an exacerbation of COPD (OR = 0.60; 95% CI: 0.39-0.93).^9^

One aspect to be noted from the present study is its design as a historical cohort, which made it possible to analyze a group of patients not immunized with pneumococcal vaccine(s). If it had a prospective design, ethical considerations should be discussed. Despite its limitations, such as exclusion of immunosuppressed patients, the missing data about comparative analysis of inhaled therapies in the groups, and the absence of etiological causes of respiratory infections related to the exacerbations, this study indicates that the groups did not differ significantly in relation to COPD exacerbations, but there was a significant difference in relation to hospitalizations. This study strengthens that this preventive measure (pneumococcal vaccination) is important in preventing a severe event in COPD, such as exacerbations leading to hospitalizations.
